# Supplementary material for: Prevalence and determinants of wasting of under-5 children in Bangladesh: Quantile regression approach
Source: PLoS One. 2022 Nov 23;17(11):e0278097. doi: 10.1371/journal.pone.0278097 (PMC9683614; doi:10.1371/journal.pone.0278097)
Supplement: S1 Appendix — (DOCX) [file pone.0278097.s001.docx]

**Appendix 1**

The QR model portrayed by the conditional $\tau$th quantiles of the outcome $Y$ for considering the values of predictors $x_{1},x_{2},...,x_{p}$ can be expressed as$Q_{y}\left(\tau\left|x_{1},x_{2},...,x_{p}\right.\right)=\beta_{0}^{\left(\tau\right)}+\beta_{1}^{\left(\tau\right)}x_{1}+...+\beta_{p}^{\left(\tau\right)}x_{p},0<\tau<1$,

where $\beta^{\left(\tau\right)}=\left(\beta_{0}^{\left(\tau\right)},\beta_{1}^{\left(\tau\right)},...,\beta_{k}^{\left(\tau\right)}\right)^{T}$is the vector of unknown parameters.

For a random sample $\left\{y_{1},y_{2},...,y_{n}\right\}$ of $Y$, it is understood that the sample median minimises the following sum of absolute deviations, . Likewise, the general $\tau$th sample quantile $\xi\left(\tau\right)$, that is the equivalent of $Q\left(\tau\right)$, is formulated as the minimiser: , where $\rho_{\tau}\left(Z\right)=Z\left(\tau-I\left(Z<0\right)\right)\text{ for }0<\tau<1$ denotes the loss function with an indicator function $I\left(.\right)$. The loss function $\rho_{\tau}$ allocates a weight of $\tau$ and $1-\tau$ for positive residuals =$y_{i}-\xi$ and negative residuals respectively. The linear conditional quantile function along with this loss function expands the $\tau$th sample quantile $\xi\left(\tau\right)$to the regression setting in the similar way that the linear conditional mean function expands the sample mean. The OLS estimates is obtained based on the linear conditional mean function $E\left(Y\left|X=x\right.\right)=x'\beta$, by solving [1].

The estimated parameter minimises the sum of squared residuals as the sample mean minimises the sum of squares . Quantile regression also estimates the linear conditional quantile function, $\left(\tau\left|X=x\right.\right)=x'\beta\left(\tau\right)$, by solving . For any quantile $\tau\in\left(0,1\right)$the quantity $\hat{\beta}\left(\tau\right)$is known as the $\tau$th regression quantile. For example $\tau=0.5$, which minimises the sum of absolute residuals, and also corresponds to $L_{1}$-type or median regression. The set of regression quantiles $\left\{\beta\left(\tau\right):\tau\in\left(0,1\right)\right\}$ is called the quantile process [1].

The QR model aimed at solving the term , where $e_{i}=y_{i}-x'_{i}\beta$ is the $i$th value of unknown errors, $\sum_{i} \tau\left|e_{i}\right|$ gives the asymmetric penalties $\tau\left|e_{i}\right|$ for over prediction and $\sum_{i} \left(1-\tau\right)\left|e_{i}\right|$ gives the asymmetric penalties $\left(1-\tau\right)\left|e_{i}\right|$for under prediction [1]. The $\tau$th quantile regression estimator $\hat{\beta}\left(\tau\right)$ is obtained by minimising the following objective function over $\beta_{\tau}$

$$Q\left(\beta_{\tau}\right)=\sum_{i\in\{i:y_{i}\geq x'_{i}\beta\}}^{N} \tau\left|y_{i}-x'_{i}\beta_{\tau}\right|+\sum_{i\in\{i:y_{i}<x'_{i}\beta\}}^{N} \left(1-\tau\right)\left|y_{i}-x'_{i}\beta_{\tau}\right|$$

where, $0<\tau<1,\text{ } i:y_{i}\geq x'_{i}\beta$ for over prediction, $i:y_{i}<x'_{i}\beta$ for under prediction [1].

**Reference**

1. Yirga AA, Ayele DG, Melesse SF. Application of Quantile Regression: Modeling Body Mass Index in Ethiopia. Open Public Health J. 2018;11: 221–233. doi:10.2174/1874944501811010221
