# Supplementary material for: Prevalence and determinants of wasting of under-5 children in Bangladesh: Quantile regression approach
Source: PLoS One. 2022 Nov 23;17(11):e0278097. doi: 10.1371/journal.pone.0278097 (PMC9683614; doi:10.1371/journal.pone.0278097)
Supplement: Attachment — Submitted filename: Response to Reviewers.pdf [file pone.0278097.s002.pdf]

Author responses to the review comments:

We would like to express our sincere gratitude to the two reviewers and the Academic Editor for their valuable comments. We have considered all the comments made by the reviewers and thoroughly revised and formatted the manuscript accordingly. A detailed response to each of the comments is provided in the table below:

**Academic Editor comments:**	**Response**	**Note**
Please include the following items when submitting your revised manuscript:<br><br>A rebuttal letter that responds to each point raised by the academic editor and reviewer(s). You should upload this letter as a separate file labeled 'Response to Reviewers'.<br>A marked-up copy of your manuscript that highlights changes made to the original version. You should upload this as a separate file labeled 'Revised Manuscript with Track Changes'.<br>An unmarked version of your revised paper without tracked changes. You should upload this as a separate file labeled 'Manuscript'.<br><br>If you would like to make changes to your financial disclosure, please include your updated statement in your cover letter. Guidelines for resubmitting your figure files are available below the reviewer comments at the end of this letter.	Thank you very much. The required files are submitted through the submission system.<br><br>We include all required information in the cover letter.	
**Journal Requirements:**	**Response**	**Note**
When submitting your revision, we need you to address these additional requirements.<br><br>1. Please ensure that your manuscript meets PLOS ONE's style requirements, including those for file naming. The PLOS ONE style templates can be found at<br><br>https://journals.plos.org/plosone/s/file?id=wjVg/PLOSOne_formatting_sample_main_body.pdf and<br><br>https://journals.plos.org/plosone/s/file?id=ba62/PLOSOne_formatting_sample_title_authors_affiliations.pdf.	Many thanks. The manuscript is revised according to PLOS ONE's style.<br><br>All necessary files are uploaded to the system of the journal.	
2. Your ethics statement should only appear in the Methods section of your manuscript. If your ethics statement is written in any section besides the Methods, please move it to the Methods section and delete it from any other section. Please ensure that your ethics statement	Thanks for raising these points. We move the ethical statement in the Methods section.	Revised texts are in red color.<br>Page: 6

is included in your manuscript, as the ethics statement entered into the online submission form will not be published alongside your manuscript.		
**Reviewer 1 comments:**	**Response**	**Note**
Hossain and colleagues examined the prevalence and determinants of wasting among children aged below 5 years in Bangladesh using quantile regression approach based on the 2017/2018 Bangladesh DHS data. This study is critical to understanding the factors associated with the indicator of wasting in the population of this children to inform sound policy decision making. I commend the authors for the application of the quantile regression in their analysis and we look forward to more of this in the literature in relation to modelling nutritional status of under-five children. However, I have some reservations of the use of wasting as the only indicator of nutritional status in this study, the arbitrary use of the tau (i.e., quantiles) values, and why the authors ignored the hierarchical structure of the DHS data in their analysis. See further comments below:	We highly appreciate this comment.	Revised texts are in red color.
Though the authors attempt to solve an important public health problem, especially in the developing countries like Bangladesh, they failed to justify the use of only wasting as a measure of nutritional status in their study, ignoring other important indicators of nutritional status of children such as stunting (indicator of long-term malnutrition) which is the highest prevalent globally and in developing nations, and underweight among others. Notably, the three commonly used indicators of nutritional status of children below 5 years are stunting, underweight and wasting. Each of this captures different dimension of under-five malnutrition so the authors must provide a scientific reason for choosing only wasting (indicator for short term malnutrition) as the only nutritional status in their study.	Thanks for your in-depth review of the manuscript and potential feedback. We appreciate these comments as they will be helpful to enhance the quality and readability of the manuscript.<br><br>The justification is added in the Introduction section.<br><br>The authors are well known about the three different dimensions used indicators of nutritional status of under-5 children. There are 45.4 million wasted children under the age of five. only more than a quarter of 194 countries are on track to meet the World Health Assembly's (WHA) 2025 target of keeping the prevalence of wasting under 5.0 percent. Moreover, it has the greatest short-term case fatality rate of any form of malnutrition.	Revised texts are in red color.<br>Page: 4
Also, like any other DHS data, the Bangladesh DHS data is hierarchical in nature where we have children nested within households, and household nested within clusters (i.e., communities) but the authors did not explain how they account for the hierarchical structure of the data used in this study. Assuming this was not explored during their modelling stage using multilevel quantile regression analysis, it could	The authors are grateful to the reviewer for highlighting these points.<br><br>We add this in the limitation section.	Revised texts are in red color.<br>Page: 17

lead to spurious statistical significance with its associated misleading interpretations. Fortunately, we currently have statistical software packages that allow easy implementation of the multilevel quantile regression analysis. Authors are encouraged to explore this and compare the results for the single level quantile regression to improve the quality of their results in the manuscript.		
Furthermore, the arbitrary use of the quantile values is not very informative in this study. Analysing nutritional status indicators using quantile regression should be guided by the thresholds for the quantiles and what they are measuring. For example, a quantile threshold between [0.01, 0.2] measures severe form of stunting, wasting and underweight. Thus, the authors should make conscious efforts to include these thresholds among the selected quantiles analysed and interpret same in relation to the severity of the nutritional status alongside other thresholds outside these to inform sound nutrition policies for these children. They considered 0.1 through 0.9 without any attention to the interpretation in relation to the severity of the wasting based on the quantile regression model. The authors will benefit from the paper by Aheto (2020) below that addressed this issue.	Thank you very much for pointing out this issue.<br><br>The Results section is revised as per your guidelines. We add the results of 0.2 quantile and we cite the suggested reference.	Revised texts are in red color.<br>Page: 7-15
Also, it will be helpful for the readers if the authors provide the plot of the quantile regression coefficients together with the coefficient plot from the ordinary least square regression to allow the comparison between the two approaches as done by Aheto (2020) presented in the reference below.<br><br>Reference<br>1. Aheto JMK: Simultaneous quantile regression and determinants of under-five severe chronic malnutrition in Ghana. BMC Public Health 2020, 20(1):644. https://bmcpublichealth.biomedcentral.com/articles/10.1186/s12889-020-08782-7	Thanks for your insightful comments. The manuscript is revised accordingly.<br><br>We add and discuss the plot of the quantile regression coefficients.	Revised texts are in red color.<br>Page: 7-15
The discussion and the conclusion look good, but the authors should consider the comments raised above to improve the quality of their manuscript.	Thanks for your positive comments. It motivates us.	
**Reviewer 2 comments:**	**Response**	**Note**
Abstract: Strength of associations needs to be reported	Thank you very much for your valuable comment and suggestions that help us improve the manuscript's quality. We have revised the Abstract section.	Revised texts are in red color.

		Page: 1
Methods:<br>Lines. Statistical Jargons on quantile regression could be put as a supplementary file.<br>Lines 162-164 should be placed in method section.<br>Line 172. Mean, not average.<br>Line 177. How did you define the outliers? Please mention it clearly somewhere.<br>Line 179. HAZ is stunting, not wasting. Is it just a typo or the authors coded data incorrectly?<br>Line 180. Same as above.<br>Line 185 to 200: Please always use the term that you studied. Malnutrition is a very wide term and the authors explored the factors of wasting only.<br>Lines 202 to 207. Should be placed in Method section under statistical analysis.<br>Lines 236-238: Why HAZ again? Please re-write the section carefully and focus on the relationship between the outcome variable and the predictors only.	Thanks. The title is revised as per your comment.<br>We move the Statistical Jargons on quantile regression in Appendix 1.<br>We move Lines 162-164 in the Methods section.<br>Line 172 is revised.<br>Line 177 is revised as per your comment.<br>Line 179-180, it was a typo. You are right. We revise it.<br>Thanks. Line 185-200 is revised as per your comment.<br>Lines 202-207 are placed in the Methods section.<br>Thanks. We revise typos in Lines 236-238.	Revised texts are in red color.<br>Page: 5-10
Discussion:<br>Lines 17-18: How are the authors so confirm about the confounding effect of infections? Did they test that? If not, it must be properly referred. Biologically, the linear growth spurt slows down with time. As a result wight-for-height becomes more stable with increasing age.<br>Lines 26-27: It was tough to get the meaning. I don't know why the authors brought the long-term malnutrition issues here?<br>Line 35. "Therefore, the government's efforts to…….''- totally redundant.<br>Line 43: "cross-protective immunity……..an enhanced innate immune response……trained immunity against"- again, totally confusing. The message is not at all clear.	We appreciate the feedback. We do not test the effect of infections. We add references in Lines 17-18.<br>Lines 26-27 are revised.<br>Line 35 is deleted because of redundancy.<br>Line 43 is deleted.	Revised texts are in red color.<br>Page: 15-16

Finally, the revised manuscript has been produced following the valuable comments and suggestions of the reviewers. Once again, we would like to thank the reviewers for their sincere dedication, professional insights, and earnest cooperation in reviewing the manuscript.
