# Supplementary material for: Prevalence and determinants of wasting of under-5 children in Bangladesh: Quantile regression approach
Source: PLoS One. 2022 Nov 23;17(11):e0278097. doi: 10.1371/journal.pone.0278097 (PMC9683614; doi:10.1371/journal.pone.0278097)
Supplement: Attachment — Submitted filename: Response to Reviewers.docx [file pone.0278097.s003.docx]

**Author responses to the review comments:**

We would like to express our sincere gratitude to the two reviewers and the Academic Editor for their valuable comments. We have considered all the comments made by the reviewers and thoroughly revised and formatted the manuscript accordingly. A detailed response to each of the comments is provided in the table below:

| **Academic Editor comments:** | **Response** | **Note** |
| --- | --- | --- |
| Please include the following items when submitting your revised manuscript:  A rebuttal letter that responds to each point raised by the academic editor and reviewer(s). You should upload this letter as a separate file labeled 'Response to Reviewers'.  A marked-up copy of your manuscript that highlights changes made to the original version. You should upload this as a separate file labeled 'Revised Manuscript with Track Changes'.  An unmarked version of your revised paper without tracked changes. You should upload this as a separate file labeled 'Manuscript'.  If you would like to make changes to your financial disclosure, please include your updated statement in your cover letter. Guidelines for resubmitting your figure files are available below the reviewer comments at the end of this letter. | Thank you very much. The required files are submitted through the submission system. |  |
| **Journal Requirements:** | **Response** | **Note** |
| Please review your reference list to ensure that it is complete and correct. If you have cited papers that have been retracted, please include the rationale for doing so in the manuscript text, or remove these references and replace them with relevant current references. Any changes to the reference list should be mentioned in the rebuttal letter that accompanies your revised manuscript. If you need to cite a retracted article, indicate the article’s retracted status in the References list and also include a citation and full reference for the retraction notice. | Many thanks. We check all the references and ensure that all are correct and complete.  All necessary files are uploaded to the system of the journal. |  |
| **Additional Editor Comments:** | **Response** | **Note** |
| 1. In the abstract, details about the data collection is not mandatory. In the manuscript, it is unnecessarily lengthy. Authors can reduce methods and materials in the abstract. | Thank you very much for your comment and feedback. We revised the abstract as per your comment. | Revised texts are in red color.  Page: 1 |
| 2. The 'Conclusion' in the abstract needs to be more specific with the results. Authors need to point which government or pubic-private organizations can make the situation better. | Thanks. The conclusion in the abstract is revised. | Revised texts are in red color.  Page: 2 |
| 3. In Table 2, authors have tested the quantiles. It is understood that if the two closer quantiles are statistically significant, their successors will be surely significant, too. In the table, two quintiles Q10 and Q20 are significant. It means the quintiles with bigger difference will be surely significant. What is the reason to include so many quintiles in the table? If authors believe that these are redundant, they may remove the additional quintile results from the table. The results are sufficiently communicated in the interpretation. | Thanks. We revised the manuscript and Remove Table 2. | Revised texts are in red color.  Page: 9 |
| 4. The authors are suggested to replace the old references with the new one. For example, a wide literature is available in recent years:  https://journals.plos.org/plosone/article?id=10.1371/journal.pone.0263470  https://www.frontiersin.org/articles/10.3389/fpubh.2022.792164/full  It provides the healthy discussion comparing different methods of measurements. | Thanks. We revised the Introduction and Discussion sections and cite the suggested papers (Ref. 10, Ref. 11). | Revised texts are in red color.  Page: 3, 16 |
| **Reviewer 1 comments:** | **Response** | **Note** |
| I thank the authors for the revision, good job.  The revision made by the authors are satisfactory. However, there are few things to correct. For example, in Table 3 column 4, the authors stated Q25 instead of Q20, leading to duplication of Q25 in the table. | We highly appreciate this comment. It was a typo. We revise it. | Revised texts are in red color.  Page: 11-13 |
| In addition, the Figure 2 provided as response to my earlier query improved the understanding of the effect of covariates on the various quantiles of weight for height z-score. However, the authors forgot to add the ordinary least squares regression coefficient line to the plot which I recommended in my earlier review to put the quantile regression in perspective. This will show why the quantile regression approach is preferred to the ordinary least squares regression approach. I provided an example based on a published paper in my first review (see below here again) to guide the authors. In that paper, the authors will find a solid red lines in Figure 2 which represent the ordinary least squares regression line. Same was stated beneath the Figure 2 in the published paper.  Reference  Aheto JMK: Simultaneous quantile regression and determinants of under-five severe chronic malnutrition in Ghana. BMC Public Health 2020, 20(1):644. https://bmcpublichealth.biomedcentral.com/articles/10.1186/s12889-020-08782-7 | Thanks for your insightful comments. Figure 2 is revised accordingly.  We also cited the suggested paper (Ref. 42). | Revised texts are in red color.  Page: 14 |
| The authors made me spent much time to review their revision because they did not do their rebuttal letter well. They are expected to respond to each item point-by-point, but in their case, they only provided responses without referring to the queries. They should remember to do this in their next revision.  Please, once the above are addressed, the paper should be sound for publication. | Thanks. We add the point-by-point author’s response file along with the revised version.  We are thankful to the reviewer for providing comments and feedback. The authors are also grateful to the reviewer for recommending publication after the revision of the manuscript. We believe that it helps to improve the quality of the manuscript. |  |

Finally, the revised manuscript has been produced following the valuable comments and suggestions of the reviewers. Once again, we would like to thank the reviewers for their sincere dedication, professional insights, and earnest cooperation in reviewing the manuscript.
